# Oleanolic Acid Initiates Apoptosis in Non-Small Cell Lung Cancer Cell Lines and Reduces Metastasis of a B16F10 Melanoma Model *In Vivo*

**DOI:** 10.1371/journal.pone.0028596

**Published:** 2011-12-09

**Authors:** Kelly Araújo Lúcio, Gleice da Graça Rocha, Leonardo Campos Monção-Ribeiro, Janaina Fernandes, Christina Maeda Takiya, Cerli Rocha Gattass

**Affiliations:** 1 Laboratório de Imunologia Celular, Instituto de Biofísica Carlos Chagas Filho, Centro de Ciências da Saúde, Universidade Federal do Rio de Janeiro, Cidade Universitária, Rio de Janeiro, Rio de Janeiro, Brazil; 2 Laboratório de Patologia Celular, Instituto de Ciências Biomédicas, Centro de Ciências da Saúde, Universidade Federal do Rio de Janeiro, Cidade Universitária, Rio de Janeiro, Rio de Janeiro, Brazil; Bauer Research Foundation, United States of America

## Abstract

**Background:**

Drug resistance, a process mediated by multiple mechanisms, is a critical determinant for treating lung cancer. The aim of this study is to determine if oleanolic acid (OA), a pentacyclic triterpene present in several plants, is able to circumvent the mechanisms of drug resistance present in non-small cell lung cancer (NSCLC) cell lines and to induce their death.

**Principal Findings:**

OA decreased the cell viability of the NSCLC cell lines A459 and H460 despite the presence of active, multidrug-resistant (MDR) MRP1/ABCC1 proteins and the anti-apoptotic proteins Bcl-2 and survivin. These effects are due to apoptosis, as evidenced by the capacity of OA to induce fragmentation of DNA and activate caspase 3. Induction of NSCLC cell death by OA cannot be explained by inhibition of the MDR proteins, since treatment with triterpene had little or no effect on the activity or expression of MRP1. Moreover, treatment with OA had no effect on the expression of the anti-apoptotic protein Bcl-2, but increased the expression of the pro-apoptotic protein Bax, altering the Bcl-2/Bax balance towards a pro-apoptotic profile. OA also decreased the expression of the anti-apoptotic protein survivin. Furthermore, OA decreased the expression of the angiogenic vascular endothelial growth factor (VEGF) and decreased the development of melanoma-induced lung metastasis.

**Conclusion:**

Our data provide a significant insight into the antitumoral and antimetastatic activity of OA in NSCLC and suggest that including OA in the NSCLC regimens may help to decrease the number of relapses and reduce the development of metastases.

## Introduction

Worldwide, lung cancer is the most frequent cause of cancer-related death [1]. Non-small cell lung cancer (NSCLC) represents over 80% of all diagnosed lung cancers. The high mortality of this disease is attributable to the difficulties of early detection. At the time of diagnosis, most patients have an unresectable, advanced disease involving lymph-node or visceral metastasis, or both. Platinum-based chemotherapy, one of the main treatments for NSCLC in the last decades, exhibited several problems: in addition to being very toxic, such chemotherapy only produced a modest improvement in overall survival [2]. Even after the development of drugs that target the growth of a tumor, the overall survival rate of patients with NSCLC remained low [3]. The chemotherapeutic failure in lung cancer may contribute to metastasis and high morbidity, indicating that effective therapies are needed.

Multidrug resistance (MDR) plays a critical role in the failure of lung cancer chemotherapy. Although several mechanisms can mediate MDR, researchers have devoted a great deal of attention to the overexpression of transporter proteins of the adenosine 5′-triphosphate (ATP)-binding cassette (ABC) family and to alterations of factors involved in the apoptotic process. Expression of MDR proteins is considered the main cause of a patient's relapse after treatment; literature data [4], [5] have described a relationship between the expression of ABC proteins and the poor outcome of NSCLC patients treated with chemotherapy. MDR proteins such as P-glycoprotein (P-gp)/ABCB1 and Multiple Drug Resistant Protein 1 (MRP1) work as efflux pumps that are capable of removing a variety of drugs from the cell, thereby preventing cell death [6]. Alterations of mechanisms that attenuate pro-apoptotic pathways and/or amplify anti-apoptotic pathways are also important factors in the development of chemotherapeutic resistance in tumors [7], [8]. Several studies showed that inhibiting anti-apoptotic proteins of the B-cell lymphoma 2 (Bcl-2) [9], [10] or inhibitor of apoptosis (IAP) families [11], [12] sensitizes NSCLC cells to chemotherapy.

Another hallmark of malignant cancer cells is their ability to establish metastasis. Since metastatic disease, rather than local tumor growth, determines the mortality of patients, targeting tumor metastasis has the highest priority in cancer therapy. A substantial body of evidence has emerged suggesting that the axis between the vascular endothelial growth factor (VEGF) receptor and the Flk-1 kinase insert domain receptor (KDR) (VEGF-Flk-1/KDR) is the dominant signal transduction pathway regulating tumor angiogenesis and metastasis [13]. However, because the main requirement for development of metastasis is the presence of live tumor cells, mechanisms involved in drug resistance, such as expression of MDR proteins and anti-apoptotic factors, have been proposed as conditions favorable to development of metastasis [14], [15].

Many compounds of natural origin are capable of modulating drug resistance. Oleanolic acid (OA), a pentacyclic triterpenoid found in a variety of plant species, presents several biologic properties, including anti-inflammatory [16], [17], hepato- and nefrotoxicity protection [18], [19], recovery of the hematopoietic system after irradiation [20] and cytotoxicity against several cancer cell lines [21], [22], [23]. In a previous study, we showed that OA is also effective against MDR erythroleukemic cells overexpressing P-gp and its sensitive counterpart [24]. The present study was performed to examine the cytotoxic effects of OA on NSCLC cells (A549 and H460) that expressed both MRP1 [25], [26] and anti-apoptotic factors [7], [8], and to investigate the effects of this triterpene on pathways involved in drug resistance and the development of metastasis.

OA induced apoptosis in both cell lines. Treatment with this triterpene decreased the expression of survivin and increased the expression of Bax, without affecting the expression of Bcl-2 or MRP1. Moreover, OA decreased the expression of VEGF and inhibited the development of melanoma-induced lung metastasis. Together, these data suggest that OA may be a good candidate for the treatment of tumors with intrinsic expression of resistance mechanisms, such as lung tumors.

## Materials and Methods

### Materials

Oleanolic Acid (OA), molecular weight 456.7, provided by Sigma Chemical Co. (Saint Louis, MO, USA), was dissolved to 10 mM of dimethyl sulfoxide (DMSO), stored at −20°C and diluted in culture medium for use. DMSO, 3-(4,5 dimethylthiozol-2-yl)-2,5-diphenyl-tetrazolium bromide (MTT), Propidium Iodide (PI) and Rhodamine 123 (Rho123) were purchased from Sigma. 5-carboxifluorescein diacetate (5-CFDA) was obtained from Calbiochem (San Diego, CA, USA). Dulbecco's modified Eagle's medium (DMEM), fetal calf serum (FCS), penicillin and streptomycin were from Life Technologies, Inc. (USA). FACS lysing solution was from BD Biosciences (San Jose, CA). Caspases-3 assay kit (CaspGlow) was from Biovision (Mountain View, CA, USA). Antibodies against Bax (clone P-19), VEGF (clone C-1) and tubulin (clone b-7) were purchased from Santa Cruz Biotechnology (Santa Cruz, CA, USA). Bcl-2 antibody (clone 124) was from DAKO (Carpinteria, CA, USA) and survivin antibody (ab24479) was purchased from ABCAM (Cambridge, MA, USA). Biotinylated anti-mouse IgG and CY3-labeled streptavidin were from Sigma. Biotinylated anti-goat IgG, anti-rabbit IgG and Vectashield® mounting medium were purchased from Vector Laboratories (Burlingame, CA, USA). 4′,6-diamidino-2-phenylindole (DAPI) and PE-labeled anti-MRP1 (QCRL-2) were from Santa Cruz Biotechnology (Santa Cruz, CA, USA). Anti-MRP1 (clone A23, Axxora, San Diego, CA), Bcl-2 (clone bcl2-100, Zymed, San Francisco, CA), anti-mouse HPR (Amersham, Arlington, IL) and anti-rabbit HPR (GE Healthcare, Piscataway, NJ) were used for the western blot.

### Cells and Culture Conditions

The human non-small cell lung cancer cell lines A549 and H460 and the murine melanoma line B16F10 were grown in Dulbecco's modified Eagle's medium (DMEM), supplemented with 10% heat-inactivated fetal calf serum (FCS), 100 U penicillin and 100 µg/mL streptomycin in disposable plastic bottles at 37°C with 5% CO_2_. Cells were sub-cultured using Trypsin-EDTA every 3–4 days.

### Cell viability assay

Cell viability was assessed using the MTT assay. Briefly, 180 µL of the lung cancer cell suspension (10^4^/cells per well) was distributed in 96-well plates and incubated for 24 h at 37°C/5% CO_2_ to allow the culture to stabilize. The cells were then treated with 20 µL of medium, various concentrations of OA (10, 25, 40 or 50 µg/mL or 21.9, 54.7, 87.6 or 109.5 µM, respectively); DMSO concentrations for each dose were used as controls. After 48 h incubation, the culture was treated with 20 µL MTT (5 mg/mL) and kept for 4 h at 37°C in the dark before being centrifuged and the supernatant discarded. The formazan produced by reduction of the MTT by viable cells was dissolved in DMSO and the optical density was measured in an ELISA reader (BenchMark, Bio-Rad, CA) at 570 nm (reference filter 630 nm). Experiments were repeated at least three times. Results were expressed as the mean ± SD of percent inhibition of cell viability. To check if high concentrations of OA would interfere with MTT readings, OA were incubated with MTT in the same conditions used for determination of cell viability. No interference was observed.

### DNA fragmentation assay

Apoptosis was assessed by cell cycle analysis using flow cytometry. After 24 h resting, the plated lung cells (2×10^4^/well) were treated with media or various concentrations of OA (10, 25, and 50 µg/mL) and incubated for another 48 h. After this time, cells were harvested, re-suspended in a hypotonic fluorescent solution (50 µg/mL PI and 0.1% Triton X-100 in 0.1% Na Citrate buffer) for 1 h, at 4°C in the dark. The cell cycle was analyzed by flow cytometry (FL-2) (FACSCalibur, Becton Dickinson, San Jose, CA) to determine the sub-G0/G1 DNA content. Sub-diploid populations were considered to be apoptotic. Data acquisition and analysis were controlled by Cellquest software, version 3.1f. Each experiment was repeated at least three times. The results were presented as representative histograms and as mean ± SD of the percentage of the DNA that was fragmented.

### Caspase activation assay

Caspase-3 activation was assayed using a commercial kit, according to the instructions of the manufacturer (Biovision, Mountain View, CA). In brief, cells plated as described in *Cell viability assay* (M&M) were incubated for 48 h with medium (control) or various concentrations of OA (10, 25 or 50 µg/mL) before being harvested, centrifuged, and suspended in caspase-3 assay solution. This assay solution contains a potent caspase inhibitor conjugated to FITC that is cell permeable, non-toxic and, irreversibly to activated caspase. After 1 h of incubation (37°C, 5% CO_2_), cells were washed twice with washing buffer and the percentage of caspase-3-activated cells was analyzed by flow cytometry (FL-1). Results were presented as histograms representative of three different experiments.

### Activity of MDR proteins

The activity of the MDR proteins was determined by the accumulation of specific substrates. Rho123 and 5-carboxyfluorescein diacetate (CFDA), a non-fluorescent molecule that is converted into the fluorescent carboxy-fluorescein (CF) by intracellular esterases, were used to measure the activity of P-gp/ABCB1 and MRP1/ABCC1, respectively [27], [28].

For each experiment, the cells (1×10^5^/well) were seeded in 24 well plates and pre-incubated for 24 h at 37°C/5% CO_2_ to allow the culture to stabilize. Cells were incubated for 30 min with medium (autofluorescence); 400 nM Rho123 or 5 µM CFDA in the presence of medium, inhibitors of P-gp (25 µM Verapamil) or MRP1 (50 µM MK-571); or the desired concentrations of OA. Then the cells were washed in PBS, harvested and kept on ice until flow cytometry analysis (FACSCalibur, Beckton-Dickinson cytometer). Results were presented as representative histograms or as the mean ± SD of arbitrary units of mean fluorescence intensity (MIF).

### Expression of MDR proteins

MRP1 expression was evaluated using flow cytometry and western blot. Cells were plated and treated for 24 h with media or OA (either 25 or 50 µg/mL). For the flow cytometry, cells were harvested, permeabilized with FACS lysing solution and incubated with PE-labeled anti-MRP1 (clone QCRL-2) for 30 minutes at 4°C. After two PBS washings, cells were re-suspended in FACS solution and the fluorescence evaluated. Results were presented as representative histograms. For western blot, whole-cell extracts were prepared by diluting the cell pellets directly in RIPA (50 mM Tris–HCl, pH 8.0, 150 mM NaCl, 0.5% Sodium deoxycholate, 0.1% SDS and 1% protease inhibitor Kit (Amersham, Arlington, IL). Equal amounts of protein were separated by SDS-PAGE and transferred to polyvinylidene difluoride (PDF) membranes. Blots were blocked for 2 h in PBS/0.05%Tween containing 5% nonfat dried milk, probed with specific antibodies to MRP1 (clone A23, 1∶1000) or tubulin (clone b-7, 1∶500) overnight and incubated with HPR-conjugated secondary antibodies (1∶2000 dilution; Amersham Biosciences, UK) for 1 hour at room temperature. Blots were developed using the enhanced chemiluminescence system (ECL, Amersham, Arlington, IL) according to the manufacturer's instructions. Band intensity was quantified using the Scion Image Software and protein expression was normalized in relation to α-tubulin.

### Immunocytochemistry assays

A459 cells (3×10^4^/well) were seeded on cover slips in 24 well plates, left to rest for 24 h and then treated with medium or 50 µg/mL OA. After 24 h incubation, the cells were washed twice with phosphate saline buffer (PBS), pH 7.4, and fixed with a 4% buffered paraformaldehyde solution containing 4% sucrose for 40 min at 4°C. After three washes in PBS, the cover slips were incubated for 30 min in a 50 mM NH_4_Cl solution, pH 8.0, and then washed again in PBS (3X). Nonspecific binding of immunoglobulins was blocked for 60 min with a PBS solution containing 5% BSA, 0.1% Triton X-100, 0.05% Tween 20 and 0.01% gelatin. The next step was to inhibit endogenous biotin using a biotin blocking kit (Vector Lab.), according to the manufacturer instructions.

After that, cells were incubated with a 1∶50 dilution of a monoclonal mouse anti-Bcl-2 antibody, 2 µg/mL polyclonal rabbit anti-Bax antibody, 2 gu/mL mouse monoclonal anti-VEGF antibody or 5 ug/mL survivin antibody, all diluted in PBS containing 3% BSA and 0.05% Tween and maintained overnight at 4°C in a humid chamber. Next, cells were washed in PBS containing 0.25% Tween 20 and incubated for 1 h with 10 µg/mL of the secondary antibody selected for that trial: biotinylated anti-goat IgG, anti-rabbit IgG or anti-mouse IgG. After that step, the coverslips were washed (3 times) with PBS/0.25% Tween and incubated for 1 h at room temperature with 10 µg/mL streptavidin conjugated to Cy3. Next, the coverslips were washed and then stained with 0.5 µg/mL DAPI for 5 min. Following two more washes, one with PBS and one in distilled water, the cover slips were mounted with Vectashield® mounting medium and observed by epi-fluorescence microscopy (Eclipse E-800, Nikon, Japan). The DAPI-stained cover slips were inspected to exclude the possibility of Mycoplasma contamination.

The quantitative analysis was performed using an image analysis system (Image-Pro® Plus 4.5, Media Cybernetics, Inc, Silver Spring, MD, USA) composed of a digital camera (Evolution, Media Cybernetics, Inc, Silver Spring, MD, USA) coupled to a fluorescence microscope. High quality images of cells were captured (2048×1536 pixels buffer), using the 40× objective lens. At least twenty fields per cover slip were counted and the percentage of immunoreactive cells was calculated from the DAPI positive cells.

The expression of Bax, Bcl-2, VEGF and survivin were also assessed by western blot using specific antibodies. Plated cells were treated with medium or 50 mg/mL OA for 24 h and whole cell extracts were prepared as described in *Expression of MDR proteins* (M&M). Proteins were separated by SDS-Page, transferred to PDF membranes and probed with specific antibodies for Bax (clone P-19, 1∶1000), Bcl-2 (clone Bcl2-100, 1∶500), VEGF (clone C1, 1∶100), survivin (clone ab469, 1∶ 1000) or tubulin (clone b-7, 1∶500). Blots were developed using the enhanced chemiluminescence system (ECL, Amersham, Arlington, IL) according to the manufacturer's instructions. Band intensity was quantified using the Scion Image Software and protein expression was normalized in relation to α-tubulin.

### Development of lung metastasis induced by B16F10 melanoma cells

All animal experimental procedures were conducted in accordance with the guidelines of the Ethics Committee for Evaluation of Animal Use in Experimentation from the IBCCF/CCS/UFRJ (CAUAP-IBCCF) and approved under the designation, Project # 1005.

On day 0, male C57BL/6 mice (10 to 12-weeks-old) were injected into their tail veins with B16F10 melanoma cells (1×10^6^ cells/animal). Tumor-cell-bearing mice were divided into four groups of five and 3 days after inoculation, treated daily for two weeks (from Monday to Friday) with 20 µL of saline, of DMSO or of OA (5 mg kg^−1^ or 10 mg kg^−1^). The dose of the triterpene for the third and fourth groups was decided by reference to a previous report [29]. The body weight of each mouse was recorded every 4 days to determine whether the treatment influenced the animal's health status. Eighteen days after inoculation of the tumor cells, the mice were killed by cervical dislocation under anesthesia with CO_2_. The lungs were removed, and two independent observers determined the number of metastatic nodules on the lungs of each mouse. Results are expressed as mean ± SD of the number of metastasis.

### Statistical analysis

Data are presented as average ± SD. Student's t-test was performed using Instat software. A value of *p*<0.05 was considered statistically significant.

## Results

### A549 and H460 cell lines express active MRP1 pump

**Figure 1:**
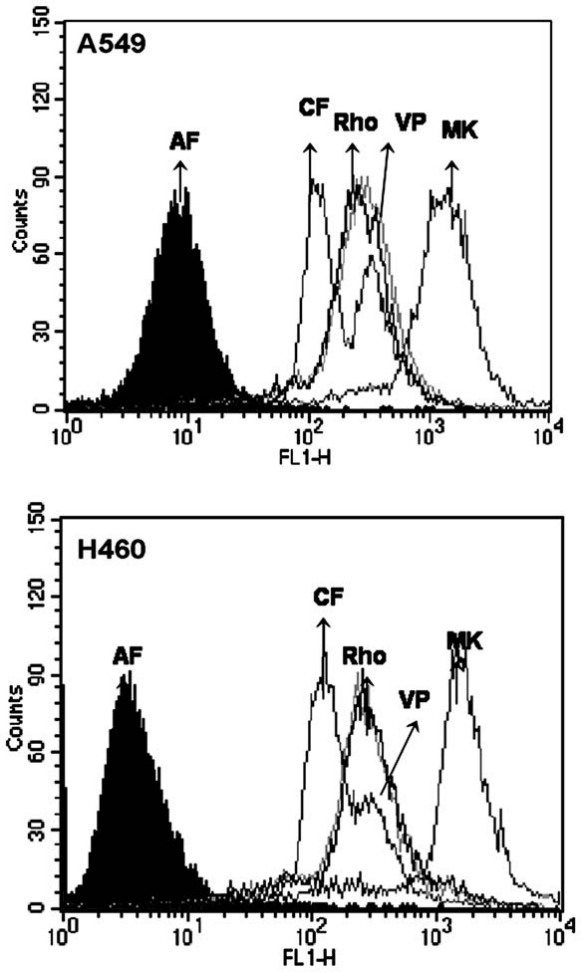
Non-small cell lung cancer cell lines express MRP1/ABCC1 activity. For P-gp/ABCB1 activity, A549 or H460 cells were incubated for 30 min with medium (AF) or 400 nM Rhodamine 123 in the absence (Rho) or presence of 50 µM verapamil (VP), For MRP1/ABCC1, cells were incubated with 5 µM CFDA in the absence (CF) or presence of 50 µM MK-571 (MK). After washing, cellular fluorescence was measured by flow cytometry. Histograms are representative of three independent experiments performed in duplicate.

The A549 and H460 cell lines express significant levels of MDR transporter proteins [25], [26]. To investigate the presence of active pumps in these lines, cells were incubated with fluorescence substrates for P-gp (Rho123) or MRP1 (CFDA) in the presence or absence of inhibitors for these proteins and then the intracellular fluorescence was measured. The lack of alteration in fluorescence observed in the presence of verapamil, a specific P-gp inhibitor [30], and the high increase in fluorescence obtained when A549 and H460 were treated with MK571, a specific MRP1 inhibitor [31], indicated that these lines have very low or no P-gp activity, but display a strong MRP1 activity (**Figure 1**).

### Oleanolic acid inhibits viability and induces caspase-dependent DNA fragmentation in NSCLC lines

**Figure 2:**
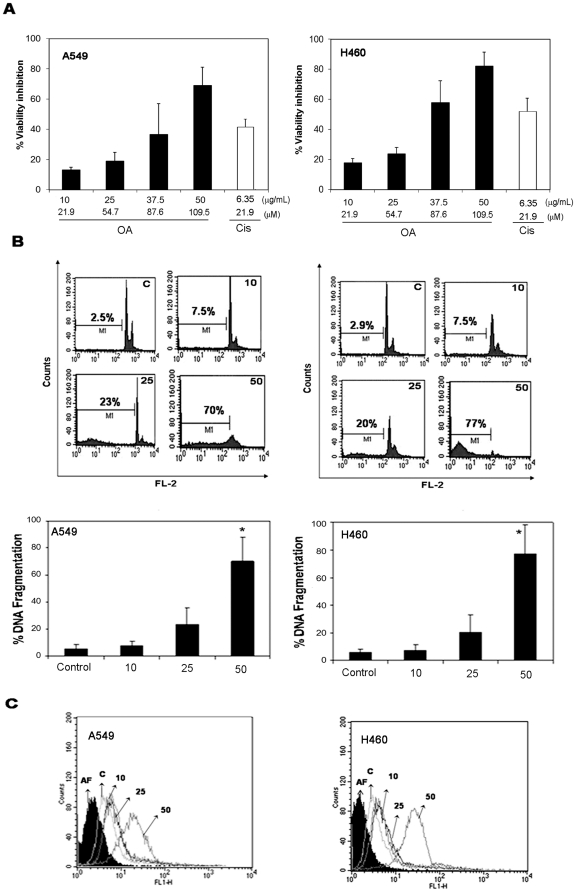
Oleanolic acid (OA) decreases cell viability and induces apoptosis of non-small cell lung cancer cell lines. A549 or H460 cells were incubated with various OA concentrations (10, 25, 40 or 50 µg/mL or 21.9, 54.7, 87.6 or 109.5 µM) or cisplatin for 48 h. (**A**) Cell viability was assessed by MTT and plotted as percentage of cell viability inhibition. Results represent mean ± SD of 4 experiments performed in triplicate. (**B**) In parallel, cells were treated with a hypotonic solution containing propidium iodide (PI) and DNA-content was analyzed by flow cytometry. Upper panel: representative histogram of the cell cycle. Lower panel: percentage of apoptotic sub-G1 cells. Values represent mean ± SD of 3 experiments performed in triplicate. (**C**) **C**aspase-3 activated cells was determined by flow cytometry using a CaspGlow kit as described in M&M. Histograms are representative of two independent experiments performed in duplicate.

To evaluate the cytotoxic effect of OA on A549 and H460, cells were treated with different concentrations of OA or with cisplatin (a traditional chemotherapeutic drug for NSCLC) and then, 48 h later; viability of the cells was measured by a MTT assay. Oleanolic acid decreased the viability of the cell lines in a dose-dependent manner (Figure 2A). Microscopic observation of the cells suggested that the effect of OA was due to apoptosis. To explore this observation further, treated cells were incubated with a hypotonic solution containing PI and then a cell cycle analysis was performed by flow cytometry (FCM). Sub-diploid populations were considered apoptotic. The increased percentage of fragmented DNA cells and the activation of caspase-3 activity induced by the OA treatment (Figures 2B and 2C), reinforced the apoptotic nature of OA cytotoxicity. This observation was confirmed by AnnexinV/PI data (data not shown).

### Effects of oleanolic acid on MDR activity and expression

**Figure 3:**
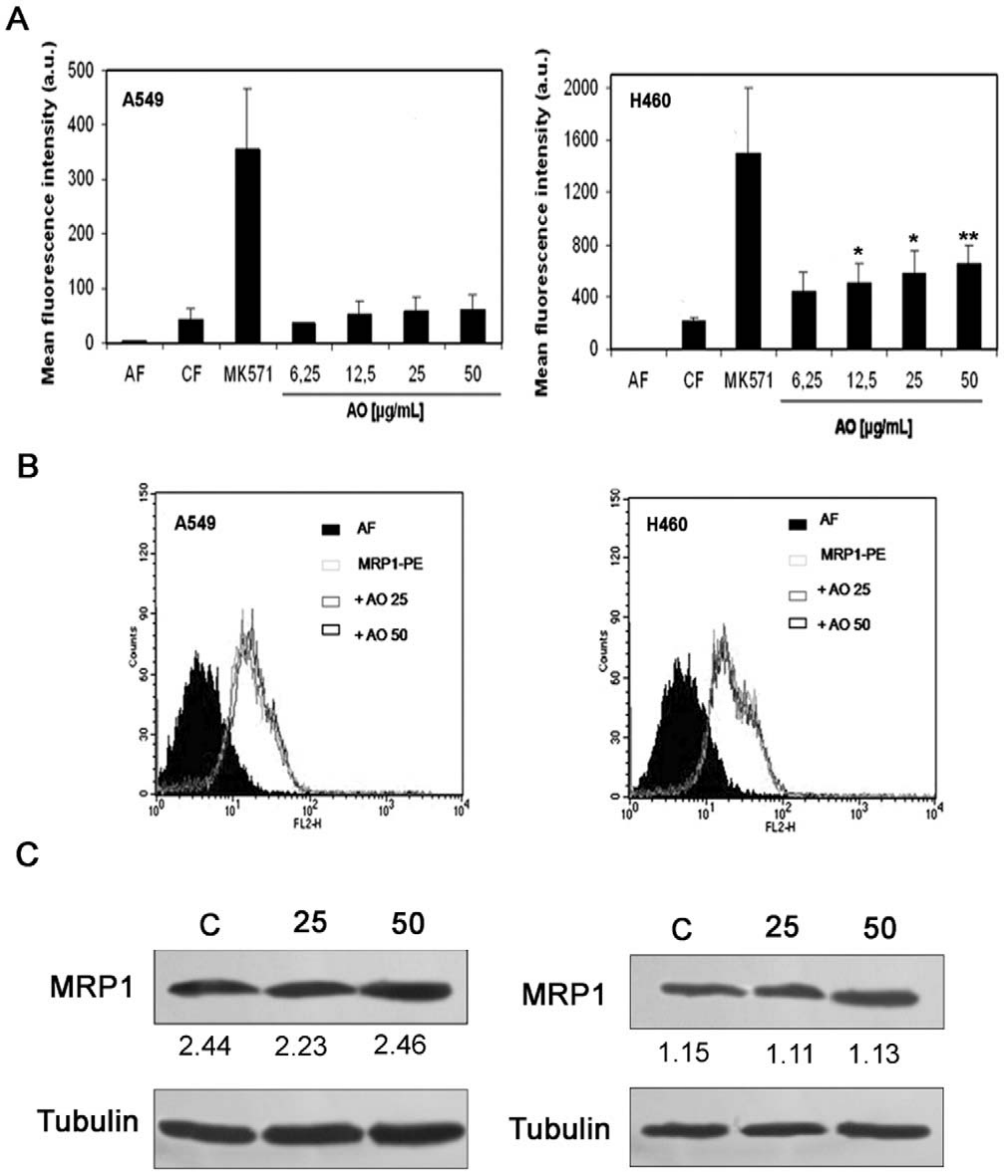
Effect of Oleanolic acid on MRP1/ABCC1 activity and expression. (**A**) To determine MRP1 activity, A549 or H460 cells were incubated for 30 min with medium (AF), 5 µM CFDA in the absence (CF) or presence of 50 µM MK-571 (MK-571), or with CFDA in the presence of various concentrations of OA (6.25, 12.5, 25 or 50 µg/mL). Cellular fluorescence was measured by flow cytometry. Results are expressed as the mean ± S.D of the mean fluorescence intensity obtained in 3 different experiments performed in triplicate. * and ** indicate *p*<0.05 and *p*<0.01 respectively, in relation to the control (CF). (**B**) To determine MRP1 expression, cells were treated with medium or OA (25 or 50 µg/mL) for 24 h before being harvested, permeabilized and incubated with PE-labeled anti-MRP1 for 30 minutes at 4°C. After two PBS washings, the cells were re-suspended in FACS solution and the fluorescence was evaluated. Histograms are representative of three independent experiments. (**C**) To determine MRP1 by western blot, whole-cell extracts were obtained from cells treated as described in B. Proteins were separated by sodium dodecyl sulphate (SDS)-gels, transferred to PDF membranes and probed with an antibody to MRP1 as described in the M&M section. Numbers represent band intensity in relation to α-tubulin.

To investigate if the cytotoxic effect of OA on A549 and H460 was mediated by modulation of the MDR pump, we analyzed the effects of OA on MRP1 activity and expression. Cells were incubated for 30 min with CFDA in the presence of different OA concentrations (6.25, 12.5, 25 or 50 µg/mL) and the accumulation of CF was measured by fluorescence. As shown in **Figure 3A**, treatment with OA did not alter the accumulation of CF by A549. A small but significant increase in fluorescence was observed in H460, suggesting that OA may be able to modulate MRP1 activity. On the other hand it was also possible, instead, that OA modifies the expression of MRP1. This possibility was analyzed by FCM and western blot in cells incubated with medium or OA (25 or 50 µg/mL, each for 24 hours and then treated with anti-MRP1. As shown in **Figures 3B and 3C**, no alteration of MRP1 expression was observed.

### Oleanolic acid inhibits the expression of proteins involved in resisting apoptosis and inducing angiogenesis on the A549 cell line

**Figure 4:**
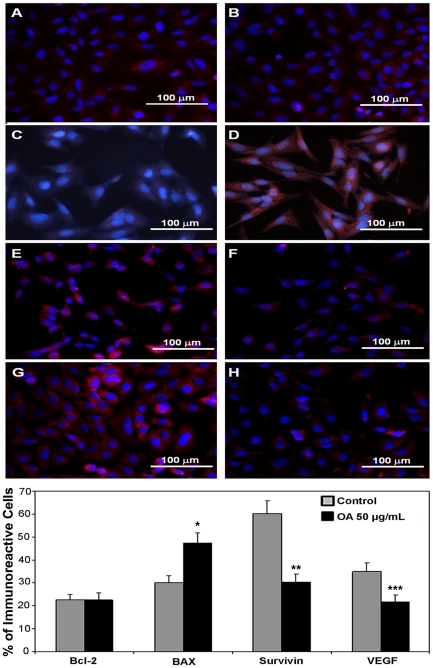
Effect of Oleanolic acid (OA) on proteins involved on apoptotic and metastatic pathways. **(Upper panel)** Immunofluorescence of A549 cells stained for Bcl2 (A–B), Bax (C–D), survivin (E–F) and VEGF (G–H). A549 cells were treated with medium (A,C,E,G) or with 50 µg/mL OA (B, D, F, H) for 24 h, fixed, stained with the primary antibodies, revealed with biotinilated IgG followed by streptavidin-Cy3 and counter stained with DAPI, as described in M&M. Representative immunofluorescence micrographs of three experiments. Bar: 100 µm. **(Lower panel)** Graphical representation of the histomorphometrical results of the immunocytochemical assay. The percentage of immunoreactive cells was calculated from the DAPI positive cells. Bcl-2 was not modulated by OA (p>0.05), but Bax was significantly increased (* *p*<0.005), while survivin and VEGF were significantly decreased after treatment (**p<0.0001 and ***p<0.05, respectively).

**Figure 5:**
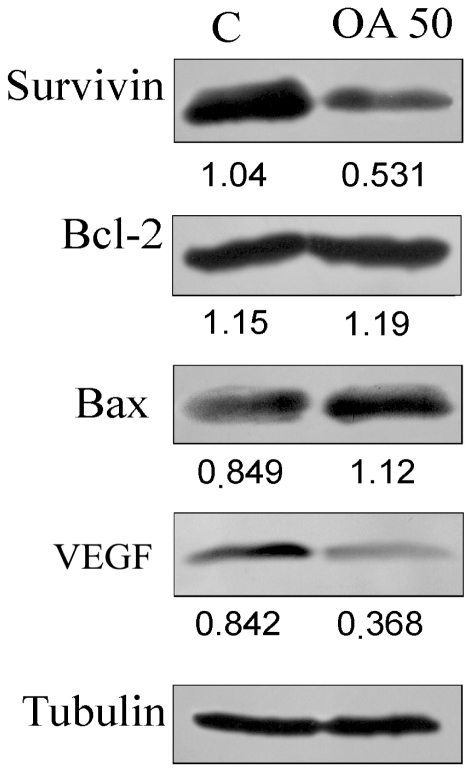
Western blot analysis. A549 cells were treated with medium or with 50 µg/mL Oleanolic acid for 24 h and whole cell extracts were obtained as described in M&M. Proteins were separated by SDS-PAGE, transferred to PDF membranes, probed with antibodies to Bcl-2, Bax, Survivin, VEGF or α-tubulin and developed with ECL, as described in M&M. Numbers represent band intensity in relation to α-tubulin.

To investigate if the cytotoxic activity of OA could be due to modulation of pathways involved in apoptosis resistance, A549 cells were treated for 24 h with 50 µg/mL of this triterpene and then the expression of Bcl-2, Bax and survivin were analyzed using immunocytochemical techniques. We found no mycoplasma in cover slips prepared for immunocytochemistry. We observed that the expression of Bcl-2 did not change after treatment with OA (**Figures 4A–B**); there was a significant (*p*<0.005) increase in Bax (**Figures 4C–D**) and a significant decrease (*p*<0.0001) in expression of survivin protein (**Figures 4E–F**). These results suggested that treatment with OA altered the cellular milieu towards an apoptotic profile and that this process may also contribute to reducing the number of metastatic foci. To explore this possibility further, the effect of OA on the expression of VEGF [13], a factor involved in cell proliferation and angiogenesis, was evaluated by immunocytochemistry. Data presented in **Figures 4 (G–H)** showed that treatment with OA led to a significant reduction (*p*<0.05) of VEGF-positive cells. Similar results were observed when the effects of OA on the expression of these proteins were analyzed by western blot (**Figure 5**). Altogether, these data reinforce a possible inhibitory effect of OA on drug resistance pathways and development of metastasis.

### Oleanolic acid inhibits the development of melanoma-induced lung metastasis

**Figure 6:**
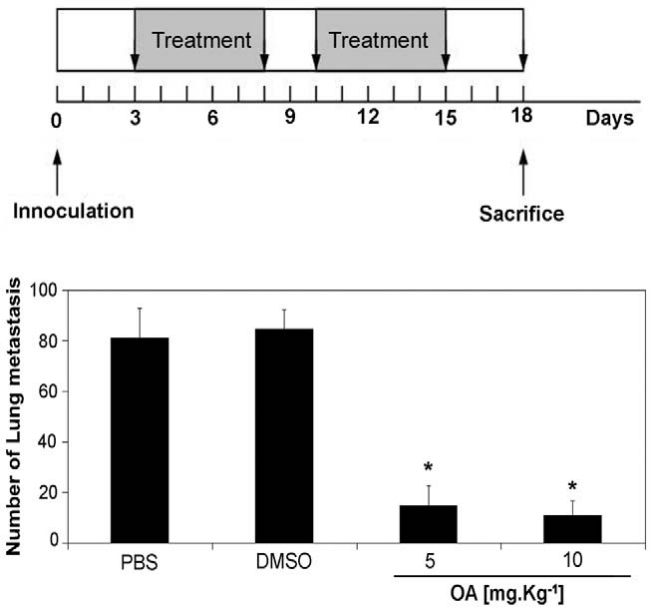
Oleanolic acid (OA) inhibits the development of lung metastasis. Groups of mice, injected into the tail veins with B16F10 melanoma cells, were treated with saline, DMSO, OA (5 mg kg^−1^ day^−1^) or OA (10 mg kg^−1^ day^−1^), as described in the M&M section and in the upper panel; the number of lung metastasis was counted on day 18 (lower panel). Results are expressed as mean ± SD of the number of metastasis. * indicates *p*<0.001 in relation to the control (DMSO).

Metastasis is generally considered a tumor growth originated from cells that lost adherence, entered the circulation, and then migrated to specific metastatic niches; in the case of lung cancer, these niches are the brain, liver and bones. In the absence of a model for metastasis specific for this particular type of lung cancer, we employed the melanoma model, frequently used in the literature to investigate the effect of drugs on metastasis, to access the effects of OA on the development of metastasis *in vivo*. For this purpose, lung metastases were induced by *i.v.* inoculation of B16F10 melanoma cells as previously described [29]. In a manner similar to A549 and H460, these cells also display an active MRP1 pump (results not shown). Starting 3 days after tumor inoculation, groups of mice were treated intra-nasally for 2 weeks with 10 doses of PBS (control) or various concentrations of OA (5 or 10 mg kg^−1^ day^−1^). The mice were sacrificed at day 17 and the number of pulmonary metastasis was counted. The higher concentration treatment with OA significantly (*p*<0.05) reduced the number of metastasis as compared to non-treated controls (**Figure 6**).

## Discussion

The presence of intrinsic or acquired resistance to chemotherapeutic agents is a major obstacle to the effective treatment of NSCLC. Indeed, the observation that about half of the patients die because the tumor spreads to distant organs has been attributed to the development of MDR. Data presented here demonstrated that, in addition to being active against NSCLC lines that expressed intrinsic MRP1/ABCC1 activity, OA modulated factors involved in apoptosis resistance and inhibited the development of melanoma-induced lung metastasis.

Because MDR proteins are found in the normal lung epithelium, tumors derived from this tissue raise levels of these proteins following chemotherapy or radiotherapy [32], [33] and are often insensitive to cytotoxic agents. Data presented in this paper demonstrated that OA is cytotoxic and induced apoptosis of two NSCLC cells that constitutively express MRP1 [25], [26] and present high MRP1 activity (**Figure 1**). Oleanolic acid-mediated cell death is due to apoptosis, as evidenced by OA's capacity to induce fragmentation of DNA and to activate caspase 3 (**Figure 2**). Although results obtained with H460 suggested that OA had a modulating effect on MRP1, no modulation was seen in A549 (**Figure 3A**). This difference is probably due to the presence of other members of the ABCC1 family of transporters in this cell line. In fact, A549 was shown to express MRP1-5 [25] and the transport properties of MRP3 and its involvement in resistance of lung cancer have been proposed [34].

Previous data from our group indicated that OA may modulate MRP1/ABCC1 activity [35]. It is important to note that despite its slight modulatory effect on MRP1/ABCC1 activity, OA did not induce an increase in the expression of MRP1 (**Figures 3B and 3C**). MRP1 is a non-selective ATP-dependent pump capable of transporting several physiological and non-physiological substrates including anticancer drugs [6]. As our group recently proposed, the capacity of OA to bypass MRP1 may be due to its physical-chemical properties, since compounds with low polar surface area such as OA are not transported by MRP1 [36]. Results showing that OA kills cells that display high MRP1/ABCC1 activity (**Fig. 2**) suggest that OA is not a substrate for this protein.

In NSCLC, drug resistance has also been associated with altered expression of anti-apoptotic members of the Bcl-2 [37] and the IAPs families [38]. Treatment with OA increased the expression of Bax without altering Bcl-2 (**Figs 4A–D** **and** **Fig. 5**), leading to a pro-apoptotic balance of these proteins in the cell. It also reduced the expression of survivin (**Figs. 4E, 4F** **and** **5**), a structurally unique member of the IAP family that plays a central role in cell division and acts as a suppressor of apoptosis [**39**]. These data suggested that OA-induced apoptosis of NSCLC may be due to a down-modulation effect of the anti-apoptotic factors present in the cell.

Increasing evidence suggests that lung cancer uses multiple and redundant pathways to maintain tumor survival and development. The results presented here showed that the ability of OA to control tumor growth is probably due to its capacity to interfere with different resistance pathways present in the cell. Thus, OA not only contributed to cell death by down-modulating anti-apoptotic factors, but it inhibited tumor cell proliferation. Since disseminated tumor cells have been found in a dormant state for prolonged periods of time [40], targeting these cells and preventing their outgrowth could be a promising approach to interfering with metastasis.

Metastasis represents the biggest problem in cancer. Metastatic relapse following systemic treatments is often associated with resistance that might be due to the cancer cell's intrinsic mechanisms, such as genetic alterations leading to expression of MDR proteins or anti-apoptotic factors [14], [41]. Treatment with OA not only bypasses these mechanisms but also significantly reduces the development of pulmonary metastasis induced by inoculation of mice with B16F10, a cell lineage that presented high MRP1 activity (data not shown) and other resistance mechanisms [42].

Drug resistance is a severe limitation on chemotherapy of various malignancies. Advanced lung cancer is a multifactorial disease that demands treatments targeting multiple cellular pathways. The search for new drugs able to overcome cancer's resistance mechanisms and to prevent the development of the metastasis favored by this disease, is of great interest for lung cancer therapy. Chemotherapy based on Cisplatin, one of the main drugs used to treat lung cancer, seemed to have reached a dead-end regarding malignant lung cancer, leading clinical researchers to evaluate the validity of continuing its use in advanced lung cancer patients.

Prolongation of survival, reduced toxicity and improved quality of life are currently the main objectives of advanced NSCLC treatment. However, because side effects were also observed in the chemotherapeutic regimens incorporating new second and third generation drugs and target-directed agents, reducing side effects may be considered an improvement over the current state of the art for lung cancer chemotherapy. In this context, OA produced its benefits without showing any hepato- or nephro-toxicity in experimental models [18], [19] and furthered the reconstitution of bone marrow after irradiation [43]. In addition, short and long term clinical trials using OA for acute and chronic hepatitis, respectively, demonstrated the safety of this compound [44].

The results presented in this paper showed that, in addition to killing cells expressing different drug resistance mechanisms, OA also attenuates the development of pulmonary metastasis. Taken together, these results indicate the potential of this compound as a chemotherapeutic agent or as a co-adjuvant for the treatment of resistant tumors such as lung tumors.
